# Draft Genome Sequence of *Alicyclobacillus acidoterrestris* Strain ATCC 49025

**DOI:** 10.1128/genomeA.00638-13

**Published:** 2013-09-05

**Authors:** Moshe Shemesh, Ronit Pasvolsky, Noa Sela, Stefan J. Green, Varda Zakin

**Affiliations:** Department of Food Quality and Safety, Agricultural Research Organization The Volcani Center, Bet-Dagan, Israela; Department of Plant Pathology and Weed Research, Agricultural Research Organization The Volcani Center, Bet-Dagan, Israelb; DNA Services Facility, University of Illinois at Chicago, Chicago, Illinois, USAc; Department of Biological Sciences, University of Illinois at Chicago, Chicago, Illinois, USAd

## Abstract

*Alicyclobacillus acidoterrestris* is a spore-forming Gram-positive, thermo-acidophilic, nonpathogenic bacterium which contaminates commercial pasteurized fruit juices. The draft genome sequence for *A. acidoterrestris* strain ATCC 49025 is reported here, providing genetic data relevant to the successful adaptation and survival of this strain in its ecological niche.

## GENOME ANNOUNCEMENT

*Alicyclobacillus acidoterrestris* is capable of surviving extremely harsh conditions, for instance during industrial food processing (1–3). *A. acidoterrestris* is a spore-forming Gram-positive bacterium, which is widespread in soil and frequently isolated from a wide variety of commodities as a contaminant (3, 4). Since it can lead to food spoilage, *A. acidoterrestris* contamination can cause enormous economic losses mainly in the fruit juice industries; therefore, this microorganism is considered a major challenge in the food industry (3, 5). *A. acidoterrestris* survives across a broad range of temperatures (25 to 60°C) and pH conditions (pH 2 to 6); it can also survive pasteurization and is able to grow during food storage (3, 5). Thus, *A. acidoterrestris* is the predominant spoilage species within the *Alicyclobacillus* genus (5). To develop our understanding of the survival strategies used by *A. acidoterrestris* in natural environments, a draft genome sequence was generated for strain ATCC 49025. The wild-type isolate of *A. acidoterrestris* ATCC 49025 was purchased from American Type Culture Collection (ATCC) and was kindly provided by Ronit Ben Avraham from Milouda Laboratories (Israel). Genomic DNA was isolated from liquid culture using a genomic DNA purification kit (Sigma-Aldrich) and prepared for shotgun sequencing using the PrepX ILM DNA library kit (IntegenX, Pleasanton, CA). DNA was initially sheared using a Covaris S2 acoustic shearing device, and subsequent to sequencing, adapter-ligated fragments were size selected (400–800 bp) using the Pippin prep automated electrophoresis instrument (Sage Scientific, Beverly, MA). Sequencing was performed on an Illumina HiSeq2000 instrument, employing paired-end 100-base reads. Approximately 13 M reads were generated in pairs and assembled by the *de novo* assembler within the software package CLC Genomics Workbench v 6.0 (CLCbio, Cambridge, MA). A total of 207 contigs of length ≥500 bases were generated, with a sum of 4,063,548 bp, an *N*_50_ of 44,524 bases, and an average coverage of >100×. More than 96% of the reads mapped to the draft genome contigs. The contigs were successfully used for annotation and gene prediction by Rapid Annotations using Subsystems Technology (RAST) (6). The overall GC content of 52.2% encompasses 4,145 predicted protein-encoding genes.

In response to stressful conditions, bacteria can initiate a developmental pathway leading to the formation of dormant endospores (7). Sporulation transcriptional activator (Spo0A) is a critical regulator for the entrance of bacteria to the sporulation pathway (8). A BLAST analysis was performed to identify sequences in the draft genome sharing high sequence similarity to Spo0A. The putative Spo0A gene in strain ATCC 49025 shows 71% similarity to the sequence encoding Spo0A in two sequenced strains of *Alicyclobacillus acidocaldarius*, Tc-4-1 and DSM 446. The *A. acidoterrestris* Spo0A protein is also similar to that of *Bacillus subtilis* 168 (61% similarity), *Bacillus licheniformis* ATCC 14580 (60% similarity), and *B. halodurans* (57% similarity). The sporulation kinase A (KinA), which activates Spo0A by phosphorylation, was also found to be conserved in *A. acidoterrestris*. Thus, KinA protein shows 32% similarity to the PAS/PAC sensor signal transduction histidine kinase of the *A. acidocaldarius* strains as well as to KinA of *B. subtilis* 168.

### Nucleotide sequence accession number.

This whole-genome shotgun project has been deposited at GenBank under the accession no. AURB00000000.

## References

[1] Connor CJ Luo H Gardener BB Wang HH 2005 Development of a real-time PCR-based system targeting the 16S rRNA gene sequence for rapid detection of *Alicyclobacillus* spp. in juice products. Int. J. Food Microbiol. 99:229–235 15808357 10.1016/j.ijfoodmicro.2004.08.016

[2] Minamikawa M Kawai Y Inoue N Yamazaki K 2005 Purification and characterization of Warnericin RB4, anti-*Alicyclobacillus* bacteriocin, produced by *Staphylococcus warneri* RB4. Curr. Microbiol. 51:22–26 15971094 10.1007/s00284-005-4456-2

[3] Steyn CE Cameron M Witthuhn RC 2011 Occurrence of *Alicyclobacillus* in the fruit processing environment—a review. Int. J. Food Microbiol. 147:1–11 21463910 10.1016/j.ijfoodmicro.2011.03.004

[4] Groenewald WH Gouws PA Witthuhn RC 2008 Isolation and identification of species of *Alicyclobacillus* from orchard soil in the Western Cape, South Africa. Extremophiles 12:159–163 17938854 10.1007/s00792-007-0112-z

[5] Spinelli AC Sant’Ana AS Rodrigues-Junior S Massaguer PR 2009 Influence of different filling, cooling, and storage conditions on the growth of *Alicyclobacillus acidoterrestris* CRA7152 in orange juice. Appl. Environ. Microbiol. 75:7409–7416 19801469 10.1128/AEM.01400-09 PMC2786409

[6] Aziz RK Bartels D Best AA DeJongh M Disz T Edwards RA Formsma K Gerdes S Glass EM Kubal M Meyer F Olsen GJ Olson R Osterman AL Overbeek RA McNeil LK Paarmann D Paczian T Parrello B Pusch GD Reich C Stevens R Vassieva O Vonstein V Wilke A Zagnitko O 2008 The RAST server: rapid annotations using subsystems technology. BMC Genomics 9:75. 10.1186/1471-2164-9-75 18261238 PMC2265698

[7] Stragier P Losick R 1996 Molecular genetics of sporulation in *Bacillus subtilis*. Annu. Rev. Genet. 30:297–241 8982457 10.1146/annurev.genet.30.1.297

[8] Molle V Fujita M Jensen ST Eichenberger P Gonzalez-Pastor JE Liu JS Losick R 2003 The Spo0A regulon of *Bacillus subtilis*. Mol. Microbiol. 50:1683–1701 14651647 10.1046/j.1365-2958.2003.03818.x

